# Survival prediction based on the gene expression associated with cancer morphology and microenvironment in primary central nervous system lymphoma

**DOI:** 10.1371/journal.pone.0251272

**Published:** 2021-06-24

**Authors:** Yasuo Takashima, Atsushi Kawaguchi, Junya Fukai, Yasuo Iwadate, Koji Kajiwara, Hiroaki Hondoh, Ryuya Yamanaka

**Affiliations:** 1 Osaka Iseikai Clinic for Cancer Therapy, Iseikai Holonics Group, Osaka, Japan; 2 Laboratory of Molecular Target Therapy for Cancer, Graduate School of Medical Science, Kyoto Prefectural University of Medicine, Kyoto, Japan; 3 Faculty of Medicine, Center for Comprehensive Community Medicine, Saga University, Saga, Japan; 4 Department of Neurological Surgery, Wakayama Medical University School of Medicine, Wakayama, Japan; 5 Department of Neurosurgery, Graduate School of Medical Sciences, Chiba University, Chiba, Japan; 6 Department of Neurosurgery, Graduate School of Medical Sciences, Yamaguchi University, Ube, Yamaguchi, Japan; 7 Department of Neurosurgery, Toyama Prefectural Central Hospital, Toyama, Japan; Hokkaido Daigaku, JAPAN

## Abstract

Dysregulation of cell morphology and cell-cell interaction results in cancer cell growth, migration, invasion, and metastasis. Besides, a balance between the extracellular matrix (ECM) and matrix metalloprotease (MMP) is required for cancer cell morphology and angiogenesis. Here, we determined gene signatures associated with the morphology and microenvironment of primary central nervous system lymphoma (PCNSL) to enable prognosis prediction. Next-generation sequencing (NGS) on 31 PCNSL samples revealed gene signatures as follows: *ACTA2*, *ACTR10*, *CAPG*, *CORO1C*, *KRT17*, and *PALLD* in cytoskeleton, *CDH5*, *CLSTN1*, *ITGA10*, *ITGAX*, *ITGB7*, *ITGA8*, *FAT4*, *ITGAE*, *CDH10*, *ITGAM*, *ITGB6*, and *CDH18* in adhesion, *COL8A2*, *FBN1*, *LAMB3*, and *LAMA2* in ECM, *ADAM22*, *ADAM28*, *MMP11*, and *MMP24* in MMP. Prognosis prediction formulas with the gene expression values and the Cox regression model clearly divided survival curves of the subgroups in each status. Furthermore, collagen genes contributed to gene network formation in glasso, suggesting that the ECM balance controls the PCNSL microenvironment. Finally, the comprehensive balance of morphology and microenvironment enabled prognosis prediction by a combinatorial expression of 8 representative genes, including *KRT17*, *CDH10*, *CDH18*, *COL8A2*, *ADAM22*, *ADAM28*, *MMP11*, and *MMP24*. Besides, these genes could also diagnose PCNSL cell types with MTX resistances *in vitro*. These results would not only facilitate the understanding of biology of PCNSL but also consider targeting pathways for anti-cancer treatment in personalized precision medicine in PCNSL.

## Introduction

The interaction of cytoskeleton with proteins involved in cancer progression or regression contributes to tumor initiation and progression, or anticancer mechanisms [1], which are associated with various signaling pathways including integrin [2], Wnt/APC [3,4], Notch [5], PI3K/AKT/mTOR [6], Ras/MAPK [7], p53 [4], and hypoxia [8]. Furthermore, various molecules and their biological functions are also required for tumor growth, such as mitotic checkpoint complex [9], cytoskeleton organization [10], cell surface morphology [11], reactive oxygen species activity [12], and ICAM-1 as a master regulator of cancer immunity and inflammation [13]. Dysregulation of intercellular connections and cell-extracellular matrix (ECM) interactions in the tumor microenvironment promotes cancer cell migration, invasion, and metastasis [14–17]. Thus, cancer cells grow in the primary lesion and spread to distant organs and lymph nodes through fluid circulation [1].

Primary central nervous system lymphoma (PCNSL) is an aggressive lymphoma of the brain with poor prognoses, which is classified as diffuse large B-cell lymphoma (DLBCL), a type of non-Hodgkin’s lymphoma (NHL) [18]. DLBCLs are divided into germinal center B-cell-like (GCB) and activated B-cell-like (ABC) types. Most PCNSLs are assigned to the non-GCB and ABC types [19]. PCNSL accounts for approximately 4% of primary brain tumors and approximately 1% of NHLs in adults [20]. The median overall survival (OS) is 30–45 months and the 5-year survival rate is 30–40% [20]. Standard treatments include high-dose methotrexate (HD-MTX)-based polychemotherapies deferred radiotherapy, but recurrence with MTX resistance is observed in most cases [21,22]. Therefore, it is necessary to determine reliable factors for prognosis prediction in PCNSL.

Although there are some similarities in morphology and molecular behaviors, microarray gene expression profiling has revealed the difference between PCNSL and non-CNS DLBCL [23]. A previous pathological study has also clarified differential expression of integrin and adhesion molecules between them [24]. In addition, a recent study has demonstrated that integrin-α, CD44, PTEN, cadherin-11, and lactoferrin as non-heme Fe^2+^-binding glycoprotein are potential biomarkers in PCNSL [25]. Hepatoma-derived growth factor, CD31, and Ki-67 are also correlated with angiogenesis, proliferation, and clinical outcome in PCNSL [26]. Moreover, a few studies have been reported on the morphology and microenvironment of PCNSL [27]. However, comprehensive analyses of PCNSL morphology and microenvironment based on gene expression profiling and statistics have not yet been performed.

In this study, we performed global expression analysis using next-generation sequencing (NGS) and multivariate analyses on 31 PCNSL samples to determine the prognostic factors associated with PCNSL morphology and microenvironment. Selected genes were further validated by combinatorial expression and survival analyses. Consequently, promising prognosis prediction factors were determined as gene signatures of cancer morphology and microenvironment, such as those related to cytoskeleton organization, cell adhesion, ECM, and matrix metalloprotease (MMP), in PCNSL. Therefore, these results would help understand the important modulator of cancer cell shapes and matrix conditions, respectively, in PCNSL.

## Materials and methods

### Clinical samples

Patients were diagnosed and treated at Toyama Prefectural Central Hospital (Toyama, Japan), Wakayama Medical University School of Medicine (Wakayama, Japan), Chiba University (Chiba, Japan), and Yamaguchi University (Ube, Yamaguchi, Japan), as described [28]. Inclusion criteria were histology-proven CNS lymphomas without the evidence of systemic lymphomas, and no evidence of HIV-1 infection, opportunistic infections, or other immunodeficiency. All tumors were derived from DLBCL in brain but not in eyes, retina, and vitreous. Of these, 24 samples were diagnosed as non-GCB DLBCL (S1 Table). The pathological characteristics including focal lesion and deep location were also presented in S1 Table. In addition, 27 samples of these were clarified copy number variations (CNVs) and cancer-associated exon mutations with the Ion Ampliseq Comprehensive Cancer Panel including representative 409 genes using the semiconductor-based Ion Proton Sequencer (Thermo Fisher Scientific) (JGAS000258). Tumor contents derived from biopsy and resected tumor tissues were more than 95%. The study was approved by the Ethics Committee of Kyoto Prefectural University of Medicine, which covered recruitments of patients from other centers (RBMR-G-146). Written informed consent was obtained from all the patients prior to enrollment. Biopsies and resected tumors were immediately snap-frozen. All experiments were performed in accordance with the institutional guidelines.

### Cells

DLBCL-type PCNSL cell lines TK and HKBML were purchased from JCRB Cell Bank (National Institutes of Biomedical Innovation, Health and Nutrition) and RIKEN Cell Bank (RIKEN BioResource Center), respectively [29]. TK is characterized in ABC-DLBCL, whereas ABC and GCB subtypes of HKBML are unknown. TK and HKBML were cultured in RPMI1640 (Nacalai Tesque) with 10% fetal bovine serum (FBS) (Thermo Fisher Scientific) and Ham’s F-12 (Nacalai Tesque) with 15% FBS, respectively, according to the standard protocol in 5% CO_2_ at 37°C. MTX-resistant PCNSL cells were generated, as described [29–32]. In brief, TK and HKBML were pre-cultured with lower concentrations of MTX for 9 weeks and 4 weeks, respectively, thereafter cultured with 1.0 × 10^−6^ mol/L MTX and 1.0 × 10^−7^ mol/L MTX, respectively, for 6 weeks. MTX-resistant PCNSL cells were kept exposing with the optimal concentration of MTX during the experiments.

### Next-generation sequencing (NGS)

Total RNA was extracted from cells and tumor biopsies or resected tissues using Isogen II (Nippongene). RNA quality was verified using the Bioanalyzer System (Agilent Technologies) with RNA Pico Chips (Agilent Technologies). NGS was performed using the Illumina HiSeq2000/2500 platform with a standard 124 bp paired-end read protocol, as described [33,34]. Genes were annotated online at GOstat [35] and Database for Annotation, Visualization, and Integrated Discovery (DAVID) [36]. Pathways were searched using DAVID and Kyoto Encyclopedia of Genes and Genomes (KEGG) [37]. The fragments per kilobase of exon per million mapped reads (FPKM) values were used for analyses (S1 Appendix).

### Survival analysis

Variable importance factors distinguishing gene expression associated with patient survival were determined by random survival forest analysis using the randomForestSRC package in R (S2 Table) [28]. The variable importance values reflected the relative contribution of each variable to the prediction of survival time, which was estimated by randomly permuting the values and recalculating the predictive accuracy of the model. Associations between the survival time of patients and other variables were evaluated with the Cox proportional-hazards regression model using the JMP built-in module (SAS Institute) [38]. Survival time distributions of the patients were calculated with the Kaplan-Meier estimator, a non-parametric statistical model based on the patient’s overall survival data, using the JMP built-in module (SAS Institute) [39]. Tree-structured survival analysis was performed to determine how the largest differences among survival curves were divided into the most appropriate subgroups with variable spaces according to the patient’s overall survival and interval censoring, using the rpart package in R [40].

### Clustering

Gene expression patterns were clustered into subgroups using a two-way hierarchical method using the JMP built-in module (SAS Institute) [38].

### Pairs plot

Pairs plot analysis was performed to estimate the distribution of single variables and relationships between two variables, using the scatterplot package in R [41].

### Graphical lasso (glasso)

Gene associations in module hub networks among variables were analyzed with the graphical lasso estimation of Gaussian graphical models, a sparse inverse covariance matrix using a lasso (L1) penalty, using the glasso package in R [42].

### Receiver operating characteristic (ROC) analysis

Samples were randomly divided into training data and test data at a ratio of 3:1. A regression equation was estimated for the training data with the Cox regression, and a time-dependent ROC analysis was carried out for the test data with the regression score and area under the curve (AUC). The 2-year and 5-year survivals were evaluated. The process was repeated 10,000 times, and the average of AUC was calculated using the timeROC package in R, as described [43].

### Statistics

Statistical analyses were performed using R, Bioconductor [44], JMP10 (SAS Institute), and Excel (Microsoft). p < 0.05 was considered statistically significant.

## Results

### Risk factors in univariate analyses

**Fig 1:**
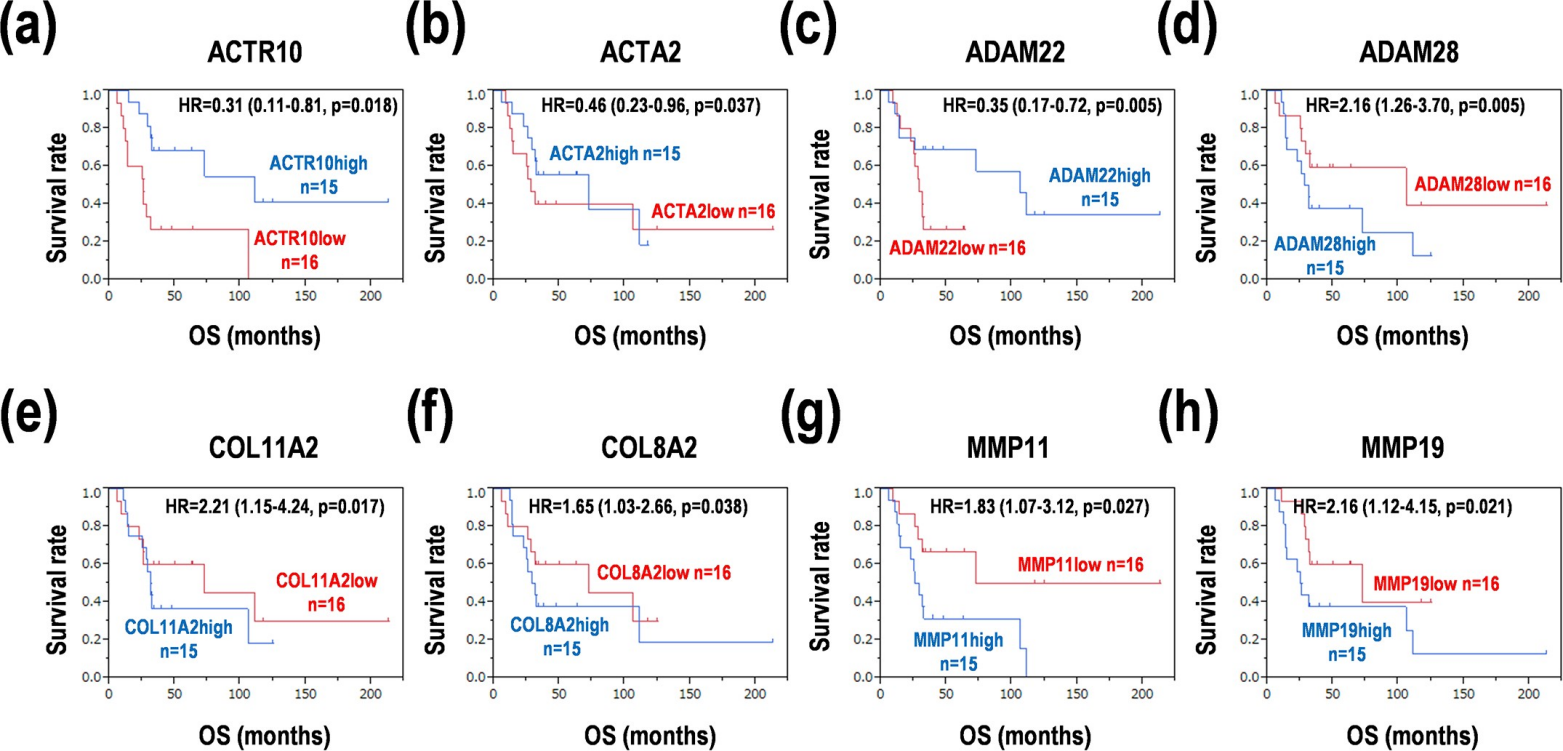
Survival distributions of the subgroups with the expression of the genes in PCNSL. (**a**) *ACTR10*. (**b**) *ACTA2*. (**c**) *ADAM22*. (**d**) *ADAM28*. (**e**) *COL11A2*. (**f**) *COL8A2*. (**g**) *MMP11*. (**h**) *MMP19*. HR; hazard ratio, OS; overall survival. Log-rank test; p < 0.05.

In this study, we tried to determine the gene signatures of cancer morphology and microenvironment in 31 PCNSL samples (S1 Table), since intercellular and cell-matrix interactions are essential for cell growth, migration, invasion, and metastasis. The 204 genes involved in cell morphology and microenvironment were selected after removals of indirect auxiliary structure genes and regulatory genes, based on Kyoto Encyclopedia of Genes and Genomes (KEGG) and gene ontology (GO). Genes associated with cytoskeleton (32 genes), cell adhesion (67 genes), ECM (39 genes), and MMP (66 genes) were constituted of clusters of several molecular types (S3 Table). A classical clustering method was difficult to divide the genes into distinct subgroups with differential expression clusters (S1 Fig). In the univariate analysis, lower expression of *ACTR10*, *ACTA2*, and *ADAM22* (Fig 1a–1c) and higher expression of *ADAM28*, *COL11A2*, *COL8A2*, *MMP11*, and *MMP19* (Fig 1d–1h) showed poor prognoses (p < 0.05). In addition, lower expression of *CDH10*, *CLSTN1*, and *MMP15* (S2a–S2c Fig) and higher expression of *COL7A1*, *ITGA10*, *ITGAL*, *ITGB7*, and *KRT17* (S2d–S2h Fig) also slightly indicated poor prognoses (p < 0.1). These results suggest that several genes associated with the cytoskeleton, cell adhesion, ECM, and MMP enable prognosis prediction in PCNSL.

### Survival prediction using multivariate analysis

**Fig 2:**
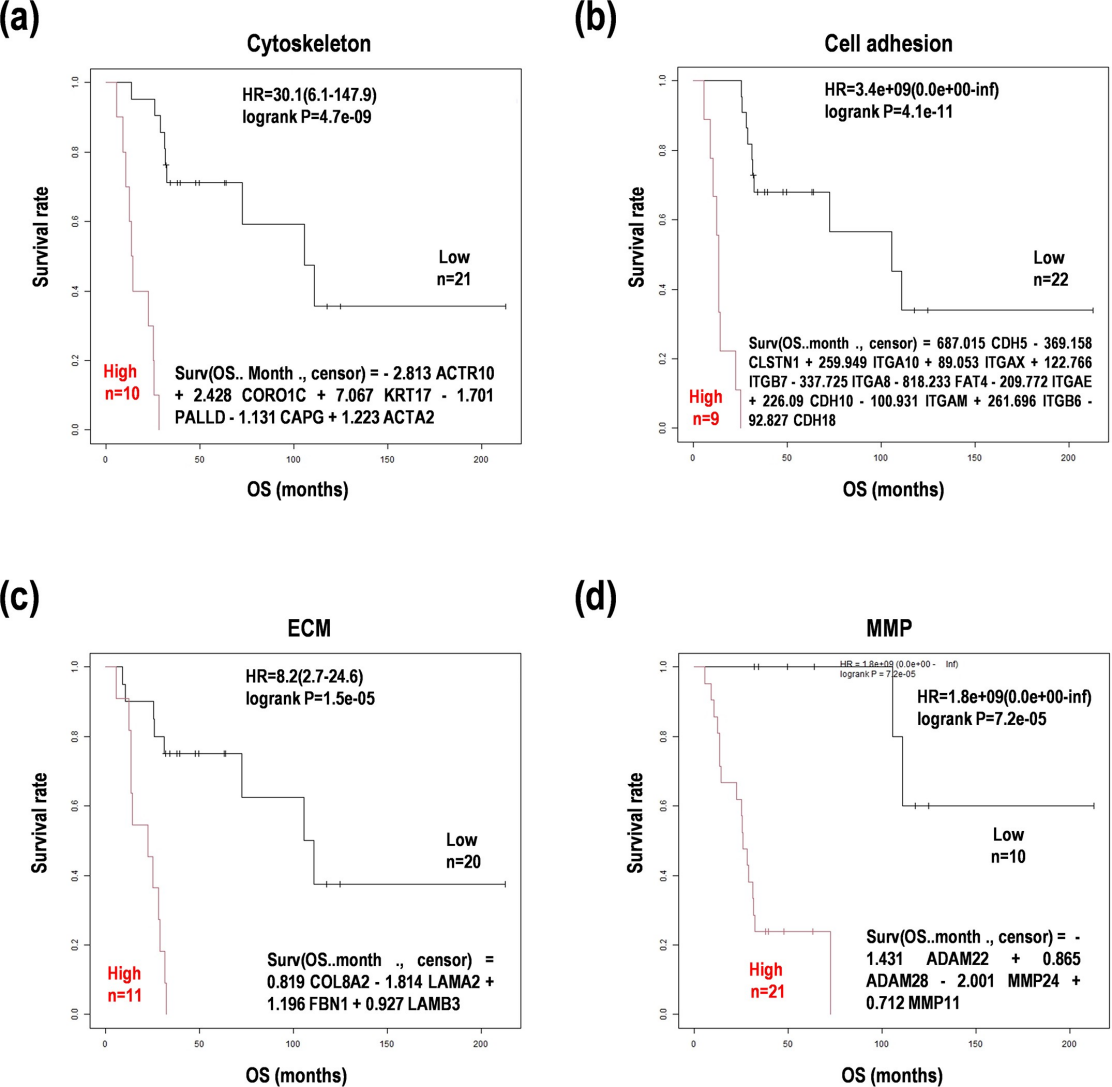
Survival analyses with defined prognosis prediction formulas. Survival distribution of the PCNSL subgroups were divided by the formulas from the study. OS; overall survival, HR; hazard ratio. HRs with 95% confidence interval (CI) were shown with p-values in the log-rank test. (**a**) Cytoskeleton. (**b**) Cell adhesion. (**c**) Extracellular matrix (ECM). (**d**) Matrix metalloprotease (MMP). HR; hazard ratio, OS; overall survival.

Next, we wanted to determine the whole balance among the genes in the subset of PCNSL samples, and then a random survival forest analysis was performed. A random forest model selected the top variables for each status (S3a–S3d Fig). Furthermore, a Cox proportional hazard regression analysis was used to estimate patient survivals (S4 Table). Coupled with these results, prognosis prediction formulas were constituted as the sum of integral of coefficient values and normalized expression values (Fig 2a–2d). The subgroups with higher scores than the median with the formulas indicated poor prognoses (p < 0.05). These results indicate that proper selection of the variable factors and their coefficients generates the prognosis prediction formulas for evaluating cancer morphology and microenvironment.

### Survival tree estimation with significant genes

**Fig 3:**
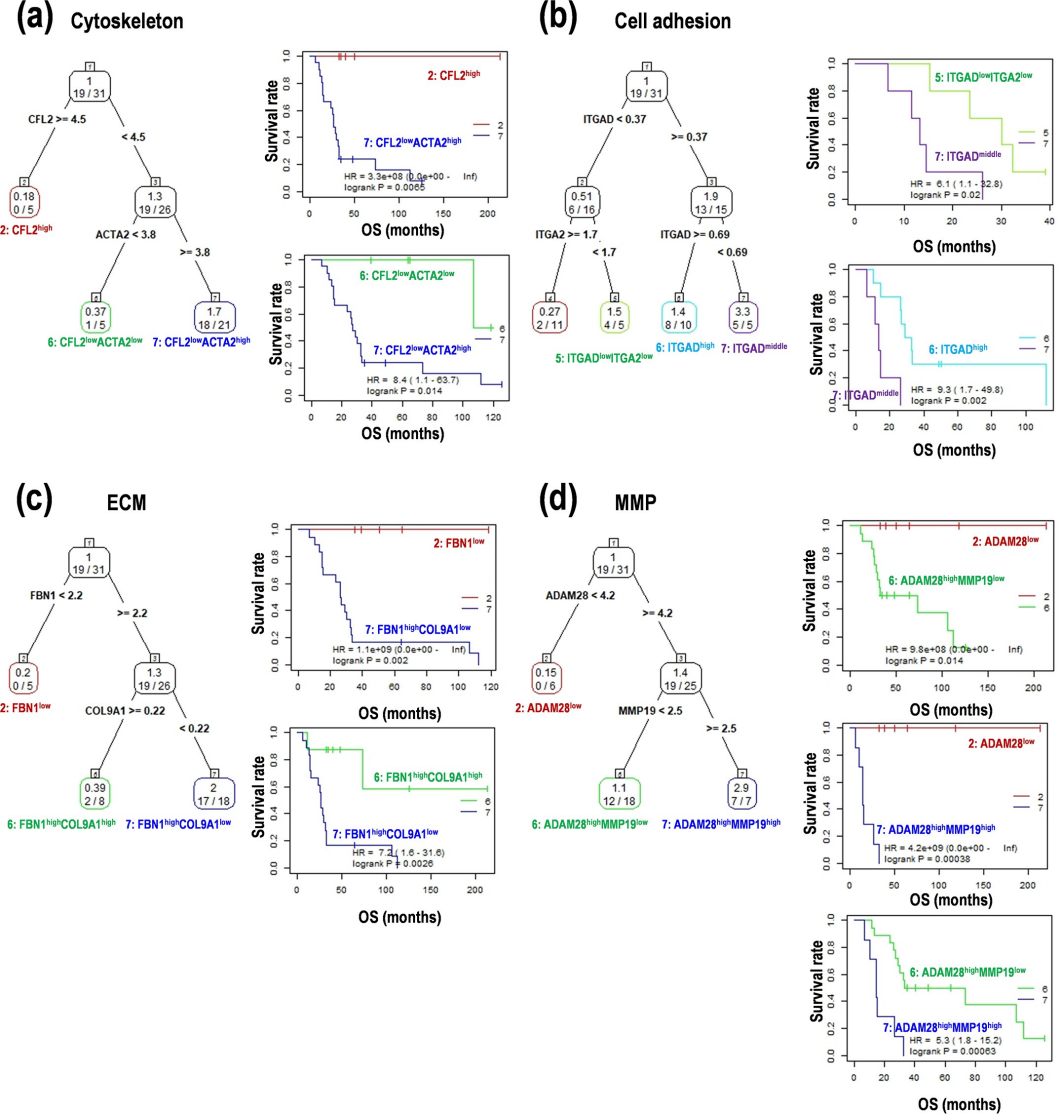
Survival regression tree model for the genes related to cancer morphology and microenvironment in PCNSL. (**a**) *CFL2* and *ACTA2* in cytoskeleton. (**b**) *ITGAD* and *ITGA2* in cell adhesion. (**c**) *FBN1* and *COL9A1* in extracellular matrix (ECM). (**d**) *ADAM28* and *MMP19* in matrix metalloprotease (MMP). Representative tree models and Kaplan-Meier curves are presented. HR; hazard ratio, OS; overall survival.

To easily predict patient survival, the significant genes in each status were examined with a survival tree analysis. In the cytoskeleton, CFL2^high^ and CFL2^low^ACTA2^low^ showed good prognoses, but CFL2^low^ACTA2^high^ indicated a poor prognosis, suggesting that lower expression of *ACTA2* is more important than *CFL2* expression for survival in PCNSL (Fig 3a). In addition, the training data set randomly divided from the total samples was also validated with the Cox regression and time-dependent receiver operating characteristic (ROC) analyses. In the internal validation, the ROC analysis returned area under the curve (AUC) 0.72 at 2-year survival and 0.81 at 5-year survival. In cell adhesion, ITGAD^low^ITGA2^low^ and ITGAD^middle^ showed good and poor prognoses, respectively. However, ITGAD^high^ showed a moderate result, suggesting that *ITGA2* expression is more significant than *ITGAD* expression (Fig 3b). In similar, the AUC values were 0.85 at 2-year survival and 0.95 at 5-year survival. In ECM, FBN1^low^ and FBN1^high^COL9A1^low^ showed good and poor prognoses, respectively, and FBN1^high^COL9A1^high^ indicated a moderate result (Fig 3c). The AUC values were 0.71 at 2-year survival and 0.78 at 5-year survival. In MMP, ADAM28^low^ and ADAM28^high^MMP19^high^ showed good and poor prognoses, respectively, and ADAM28^high^MMP19^low^ indicated moderate results, suggesting that lower expression of *ADAM28* basically determines survival in PCNSL (Fig 3d). The AUC values were 0.82 at 2-year survival and 0.82 at 5-year survival. Summarized these results, the tree-structured survival analysis and ROC analysis demonstrated that the significant genes are involved in OS in PCNSL.

### Comprehensive analysis of morphology and microenvironment status

**Fig 4:**
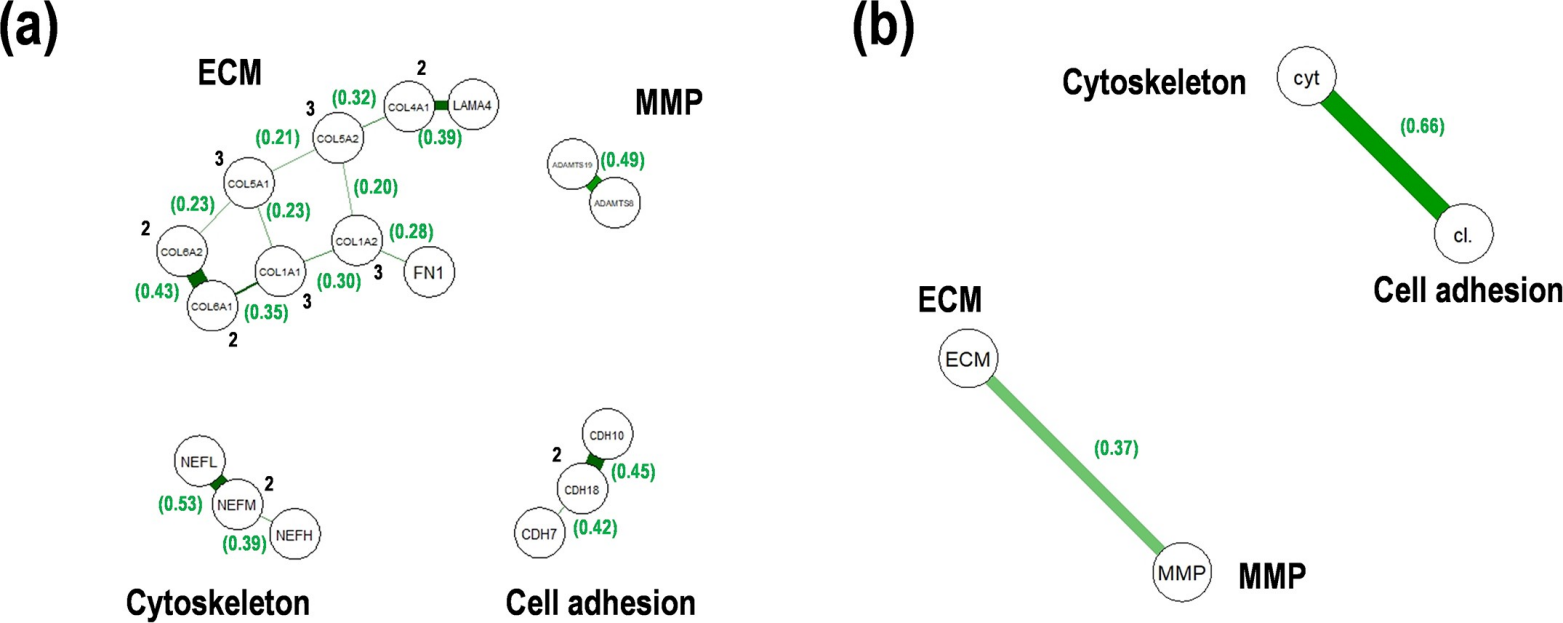
Gene correlations with graphical lasso model in PCNSL. (**a**) Score correlations within each status including cytoskeleton, cell adhesion, extracellular matrix (ECM), and matrix metalloprotease (MMP). (**b**) Score correlations among the statuses. Numbers in the parentheses indicated the edge weights of the nodes.

**Fig 5:**
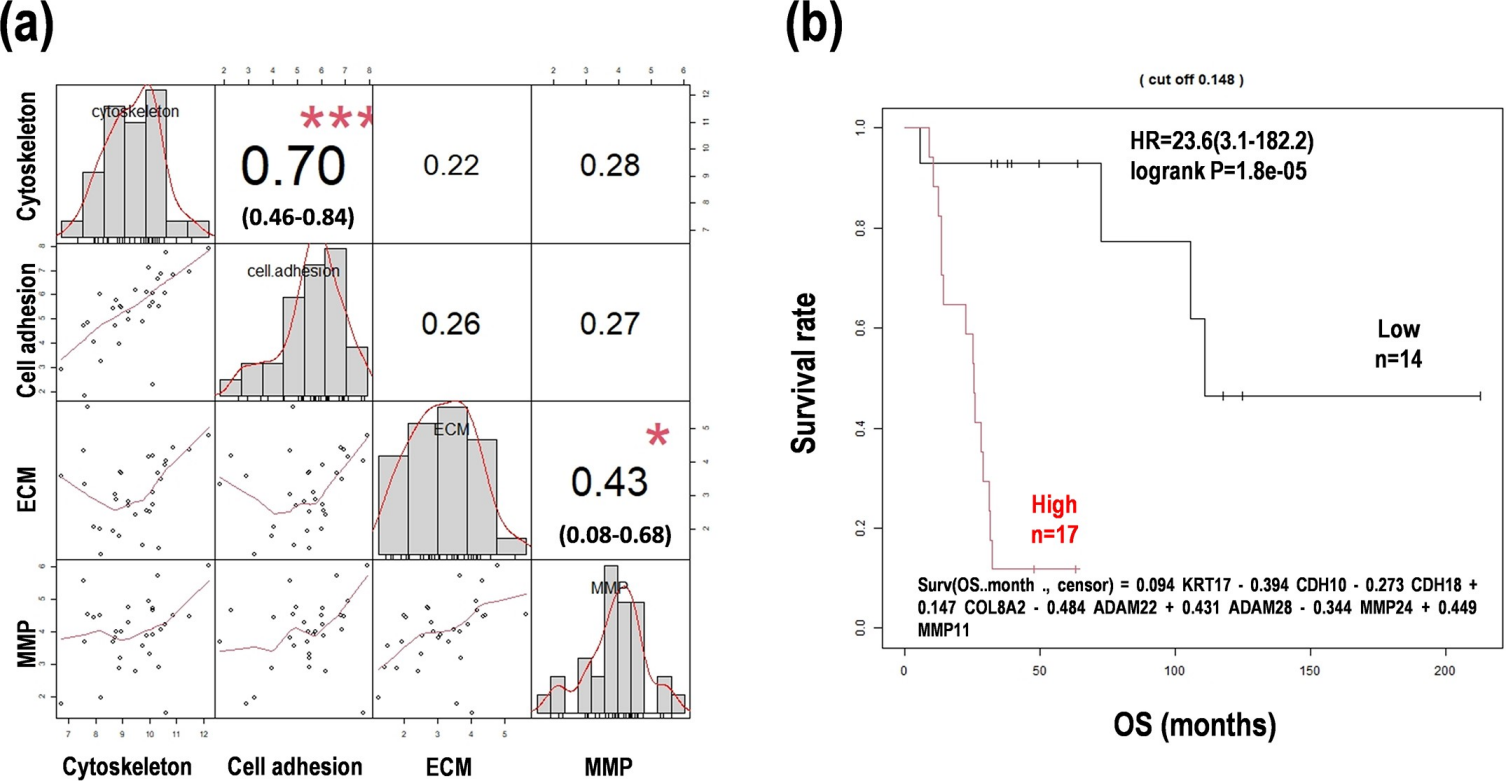
Multiscore-based survival prediction model for the statuses of cancer morphology and microenvironment in PCNSL. (**a**) Status score correlation with Pearson correlation coefficient among the statuses of cytoskeleton, cell adhesion, extracellular matrix (ECM), and matrix metalloprotease (MMP). The 95% CIs are presented in the parentheses. ***p < 0.01, *p < 0.1 (**b**) The Kaplan-Meier analysis from the multiscore-based survival prediction formula.

Genetic interactions as module hub networks among the genes were estimated using a graphical lasso model in each status. A few genetic interactions were found in the cytoskeleton, cell adhesion, and MMP (Fig 4a). On the other hand, ECM-related genes comprised a complex genetic interaction module (Fig 4a). Thus, these results suggest that it is difficult to assess the status of cancer morphology and microenvironment. In addition, status correlation analysis revealed that the cytoskeleton-cell adhesion correlation (edge weight = 0.66) and the ECM-MMP correlation (edge weight = 0.37) were distinguished, whereas no connection between the two correlations was observed (Fig 4b). Status score correlation was also analyzed in the pairs plot with Pearson correlation coefficient (r). Cytoskeleton and cell adhesion were correlated (r = 0.70, p = 6.39 × 10^−5^), ECM and MMP were slightly correlated (r = 0.43, p = 0.079), and the others were weakly correlated with no significance (r < 0.28, p = 0.53) (Fig 5a), consistent with the glasso results.

We also constructed an improved formula as a combined status formula using the four statuses for prognosis prediction. The subgroup with a higher score than the median calculated with this formula clearly indicated a poor prognosis (HR = 23.6, 95% CI = 3.1–182.2, p = 1.8 × 10^−5^) (Fig 5b). The results demonstrated that multivariate analyses using the gene expression values in cancer morphology and microenvironment generated an advanced formula and thereby enabled prognosis prediction in PCNSL, whereas it was hard to detect glasso modules and status correlations.

### Differential expression of the gene signature candidates in MTX-resistant PCNSL cells

**Fig 6:**
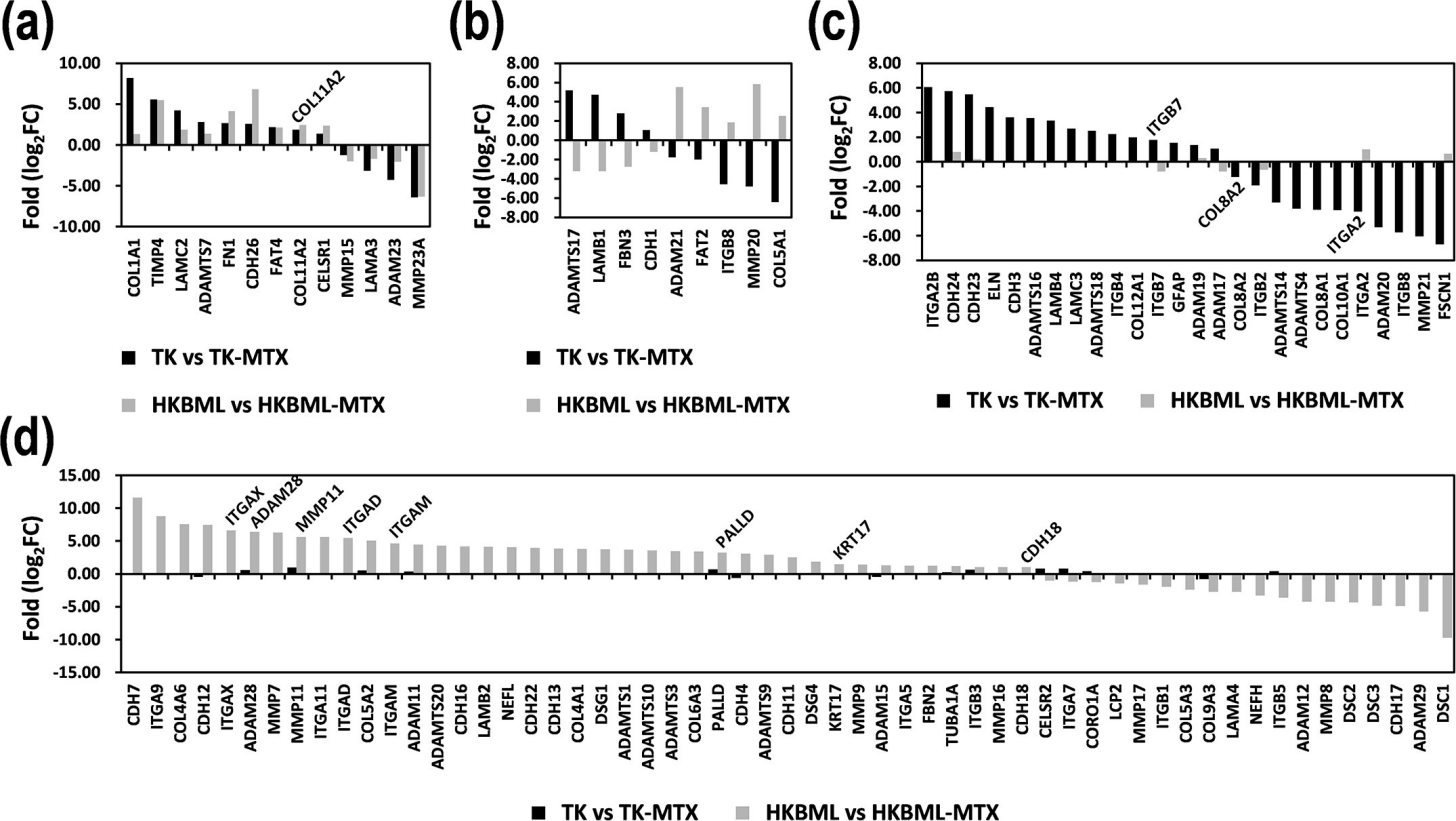
Expression patterns of the genes associated with cell morphology and microenvironment in MTX-resistant PCNSL cells. (**a**) Similar pattern of the differential expression in TK-MTX and HKBML-MTX compared with the control cells. (**b**) Reciprocal pattern of the differential expression in TK-MTX and HKBML-MTX compared with the control cells. (**c-d**) Cell-type specific differential expression in (**c**) TK-MTX and (**d**) HKBML-MTX. Gene expression was verified with NGS. DEGs in MTX-resistant PCNSL cells compared with control PCNSL cells were designated by |log_2_FC| > 1. The gene symbols highlighted into graphs contributed to prognosis prediction formulas and Kaplan-Meier survival estimation (see S5 Table). DEG; differential expression gene, FC; fold change, TK-MTX; MTX-resistant TK, HKBML-MTX; MTX-resistant HKBML.

The resistances to MTX therapies are serious problems to substantially affect the PCNSL prognosis. In addition, tumor microenvironment has a critical role in the acquisition of refractoriness to chemotherapies. Hence, comprehensive analyses in morphology and microenvironment-related genes are of extreme interest and useful for the development of prognosis factors and the understanding of PCNSL. Therefore, we investigated expression changes of the gene signature candidates associated with cancer morphology and microenvironment by NGS in MTX-resistant PCNSL cells *in vitro*. The two MTX-resistant PCNSL cell lines, TK-MTX and HKBML-MTX, derived from TK and HKBML, respectively, were examined for the 204 genes focused on this study. Differential expression genes (DEGs) in MTX-resistant PCNSL cells compared with control PCNSL cells were designated by |log_2_(fold change)| > 1. DEGs were divided into the four patterns. Thirteen genes indicated similar expression patterns in TK-MTX and HKBML-MTX (Fig 6a), and nine genes showed reciprocal expression patterns in each (Fig 6b). Interestingly, the genes with cell-type specific differential expression were 26 and 57 in TK-MTX and HKBML-MTX, respectively (Fig 6c and 6d), which could be suggestive of clonal expression. Simultaneously, it also suggests a possibility that appropriate marker set for morphology and microenvironment enables a personalized precision medicine, tailored medicine in PCNSL.

Furthermore, the differential expression in the MTX-resistant PCNSL cells was compared with the results of the above-mentioned survival analyses and construction of prognostic prediction formulas. The high expression of *COL11A2* in TK-MTX and HKBML-MTX showed poor prognoses in PCNSL (Fig 6a, S5 Table). The high expression of *ITGB7* and the low expression of *ITGA2* in TK-MTX showed poor and good prognoses, respectively (Fig 6c, S5 Table). The high expression of *ADAM28*, *MMP11*, and *KRT17* in HKBML-MTX showed poor prognoses (Fig 6d, S5 Table). The low expression of *ITGAD* indicated a good prognosis (Fig 6d, S5 Table). Furthermore, *ADAM28*, *ITBG7*, *KRT17*, and *MMP11* were also applied to the prognosis prediction formulas (Fig 6d, S5 Table). However, the low expression of *COL8A2* in TK-MTX and the high expression of *CDH18* in HKBML-MTX were not consistent with survival estimation and the input to prognostic prediction formulas (Fig 6c, S5 Table). Therefore, *COL8A2* and *CDH18* could diagnose PCNSL cell-types, such as TK-type or HKBML-type, and would be prognosis marker candidates, but not pivotal factors with MTX resistances in PCNSL.

## Discussion

Immunohistochemistry and immunoelectron microscopy in PCNSL, secondary CNSL, and systemic DLBCL have demonstrated that *ITGA10*, *CD44*, *PTEN*, *CDH1* (E-cadherin), *CDH2* (N-cadherin), *CDH3* (P-cadherin), *CDH11*, *CDH12*, and *LTF* (lactoferrin) are considered potential biomarkers of CNS tropism in adhesion, migration, and inflammatory response [25]. Perivascular lymphocytes reside within the reticulin network, which is immunopositive for collagen types III and IV, laminin, and fibronectin. These matrix components collaborate with invasion of malignant lymphocytes to CNS lymphoma in response to identical spreading mechanisms in both primary and metastatic lymphomas [45]. Therefore, adhesion molecules and ligands participate in the spreading of malignant lymphocytes within the CNS parenchyma. The expression of *FN1*, in addition to *LMO2*, *BCL2*, *BCL6*, *CCND2* (cyclin D2), and *SCYA3* is effective in estimating OS using polymerase chain reaction and microarray in DLBCL [46]. In addition, *laminin receptor 2* is a PCNSL-specific gene detected by principal component analysis on DNA arrays in a small sample size [47]. Furthermore, genes with altered promoter DNA methylation can be used as biomarkers for cancer detection and assessment of prognosis. The promoter methylation of *FBN1* is observed at 23% in NHL [48]. Although PCNSL was treated with HD-MTX-based chemotherapies, most cases recur with MTX resistance [21,22]. *MMP19* is highly expressed in the MTX-resistant PCNSL cell lines, HKBML-MTX and TK-MTX *in vitro* [29–32].

In this study, the gene expression associated with cancer morphology and microenvironment was assessed using univariate and multivariate analyses of the RNA-seq data and clinical information in PCNSL. Univariate analysis detected a correlation between differential expression of the genes related to cancer morphology and microenvironment and patient survivals with poor progoses in PCNSL. From the random forests and Cox hazard analyses, a combinatorial expression of the significant 8 genes, including *KRT17*, *CDH10*, *CDH18*, *COL8A2*, *ADAM22*, *ADAM28*, *MMP24*, and *MMP11*, selected at mixed statuses, clearly divided survival curves of the subgroups in the Kaplan-Meier estimation. A glasso model revealed genetic interaction with module hub networks. However, status correlation analyses with glasso and pairs plot did not connect the morphology status with the microenvironment status. Interestingly, survival tree analyses demonstrated poor prognoses of the subgroups with CFL2^low^ACTA2^high^ (cytoskeleton), FBN1^high^COL9A1^low^ (ECM), and ADAM28^high^MMP19^high^ (MMP). The result of ADAM28^high^MMP19^high^ was consistent with the results of ADAM28^high^ and MMP19^high^ in univariate analysis, suggesting that it is important to estimate OS with combinatorial expression of the determinant factors in multivariate analysis. Moreover, the tree analysis also proposed the possibility that *COL9A1* would be a stabilizer for *FBN1* and that *MMP19* would be an accelerator for *ADAM28*, to modulate the expression of the determinant genes for PCNSL survival. Besides, a part of genes examined were also suggestive of clonal expression in MTX-resistant PCNSL cells including TK-MTX and HKBML-MTX, and personalized diagnosis and precision medicine, so called tailored medicine in PCNSL. Therefore, these results suggest a possibility that immunohistochemistry of the cell morphology and ECM proteins on the clinical samples could be applied diagnosis of PCNSL cell-types, such as TK-type or HKBML-type, and prognosis prediction in the PCNSL patients. These results are limited due to the small sample size and the status of interest, but the results described above would help understand cancer morphology and microenvironment with patient survival and develop *de novo* molecular target therapy in PCNSL.

## Supporting information

S1 Fig Gene expression clustering in tumor morphology and microenvironment of PCNSL. Heat maps were drawn with the two-way clustering method. Gene expression with IQR > 0.1 were enrolled. (**a**) Cytoskeleton. (**b**) Cell adhesion. (**c**) Extracellular matrix (ECM). (**d**) Matrix metalloprotease (MMP). Numbers in the parentheses indicated the numbers of the genes.
(PDF)

S2 Fig Survival distributions of the subgroups with the expression of the genes in PCNSL. (**a**) *CDH10*. (**b**) *CLSTN1*. (**c**) *MMP15*. (**d**) *COL7A1*. (**e**) *ITGA10*. (**f**) *ITGAL*. (**g**) *ITGB7*. (**h**) *KRT17*. HR; hazard ratio, OS; overall survival. Log-rank test; p < 0.1.
(PDF)

S3 Fig Variable importance of the genes related to tumor morphology and microenvironment of PCNSL. Random forests survival analyses selected top variables in each category. Top variable selected were shown in graph. (**a**) Cytoskeleton. (**b**) Cell adhesion. (**c**) Extracellular matrix (ECM). (**d**) Matrix metalloprotease (MMP). The gene symbols with the top variables by the random forests survival analysis are presented in the graphs.
(PDF)

S1 Table Characteristics of the patients with PCNSL enrolled in the study. (PDF)

S2 Table Information for the R version and attached packages used in the study. (PDF)

S3 Table A list of the genes analyzed in the study. (PDF)

S4 Table Multivariable risk factors from cox hazard model in tumor morphology and microenvironment of PCNSL. (PDF)

S5 Table Summary of the gene signature candidates associated with cancer morphology and microenvironment and the differential expression with MTX resistance in PCNSL. (PDF)

S1 Appendix (XLSX)
