# Supplementary material for: Survival prediction based on the gene expression associated with cancer morphology and microenvironment in primary central nervous system lymphoma
Source: PLoS One. 2021 Jun 24;16(6):e0251272. doi: 10.1371/journal.pone.0251272 (PMC8224980; doi:10.1371/journal.pone.0251272)
Supplement: S1 Fig — Heat maps were drawn with the two-way clustering method. Gene expression with IQR > 0.1 were enrolled. (a) Cytoskeleton. (b) Cell adhesion. (c) Extracellular matrix (ECM). (d) Matrix metalloprotease (MMP). Numbers in the parentheses indicated the numbers of the genes. (PDF) [file pone.0251272.s001.pdf]

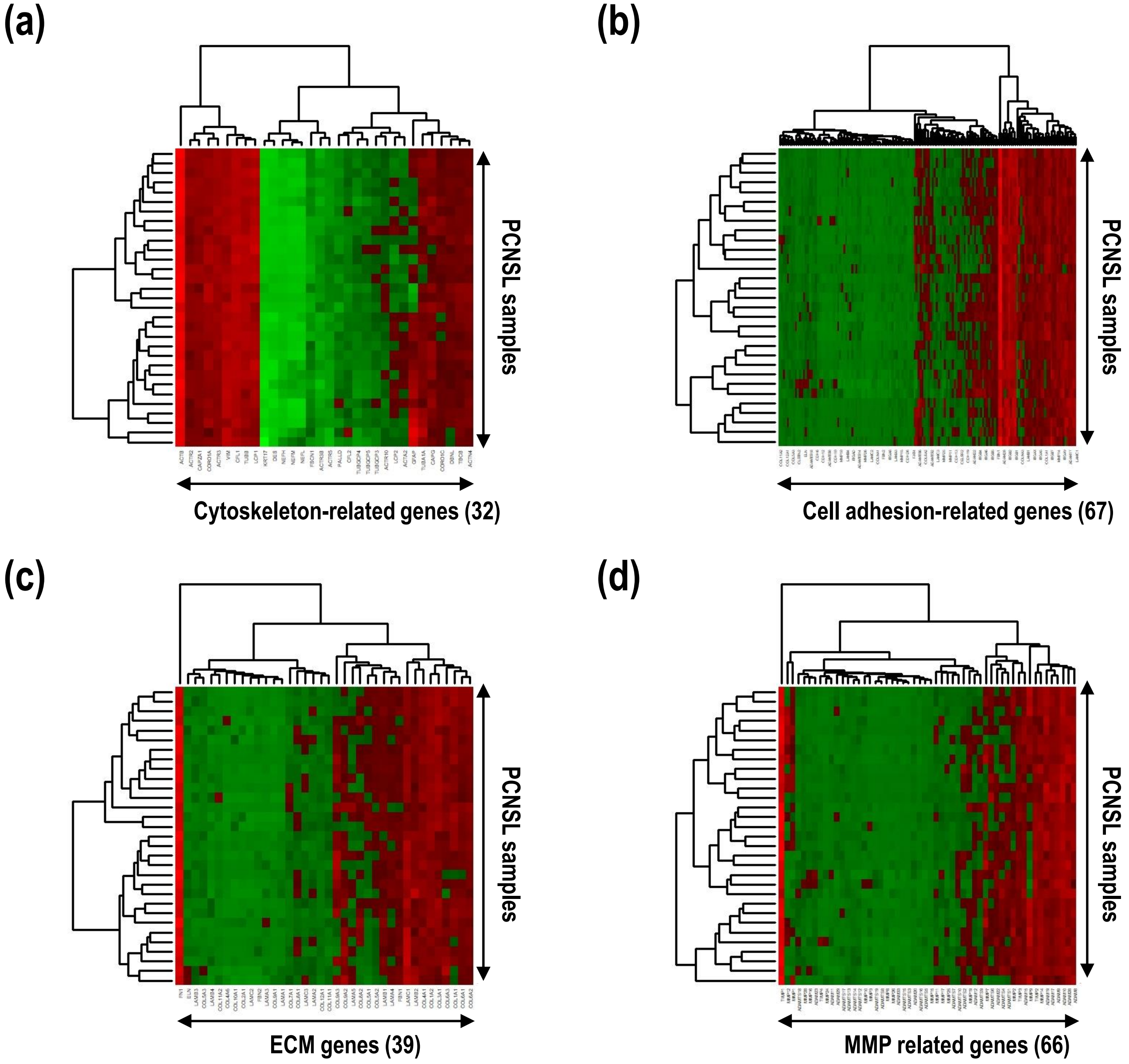

S1 Fig. Gene expression clustering in tumor morphology and microenvironment of PCNSL. Heat maps were drawn with the two-way clustering method. Gene expression with IQR>0.1 were enrolled. (**a**) Cytoskeleton. (**b**) Cell adhesion. (**c**) Extracellular matrix (ECM). (**d**) Matrix metalloprotease (MMP). Numbers in the parentheses indicated the numbers of the genes.
