# Supplementary material for: Survival prediction based on the gene expression associated with cancer morphology and microenvironment in primary central nervous system lymphoma
Source: PLoS One. 2021 Jun 24;16(6):e0251272. doi: 10.1371/journal.pone.0251272 (PMC8224980; doi:10.1371/journal.pone.0251272)
Supplement: S2 Fig — (a) CDH10. (b) CLSTN1. (c) MMP15. (d) COL7A1. (e) ITGA10. (f) ITGAL. (g) ITGB7. (h) KRT17. HR; hazard ratio, OS; overall survival. Log-rank test; p < 0.1. (PDF) [file pone.0251272.s002.pdf]

S2 Fig

Takashima et al

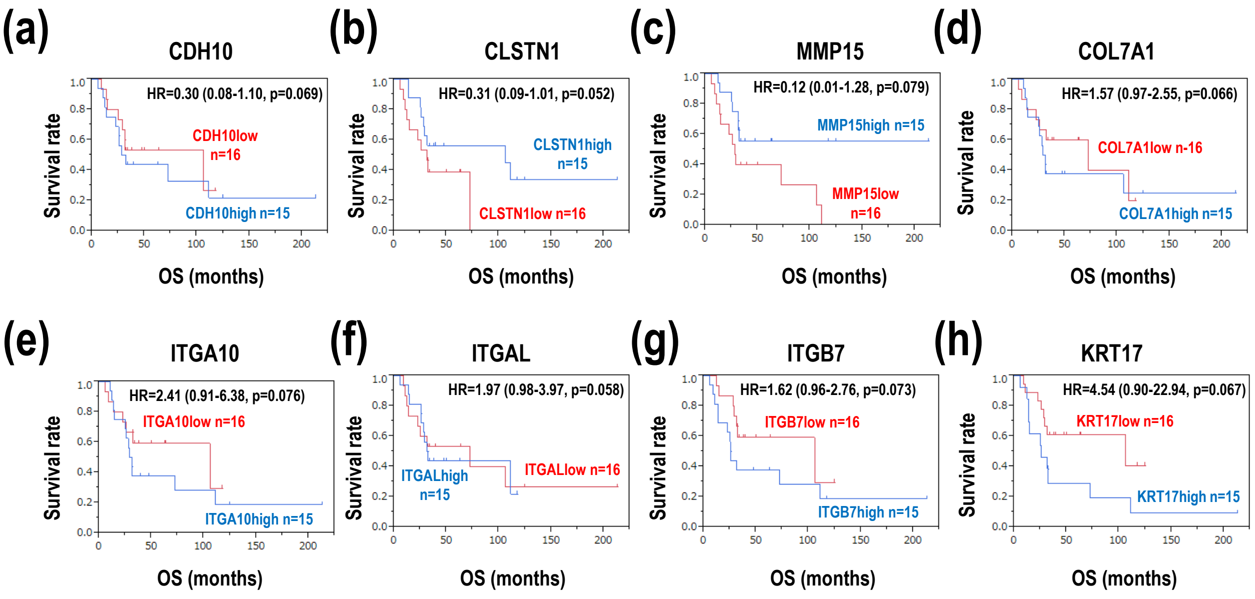

S2 Fig. Survival distributions of the subgroups with the expression of the genes in PCNSL. (**a**) CDH10. (**b**) CLSTN1. (**c**) MMP15. (**d**) COL7A1. (**e**) ITGA10. (**f**) ITGAL. (**g**) ITGB7. (**h**) KRT17. HR; hazard ratio, OS; overall survival. Log-rank test; p<0.1.
