# Supplementary material for: Survival prediction based on the gene expression associated with cancer morphology and microenvironment in primary central nervous system lymphoma
Source: PLoS One. 2021 Jun 24;16(6):e0251272. doi: 10.1371/journal.pone.0251272 (PMC8224980; doi:10.1371/journal.pone.0251272)
Supplement: S3 Fig — Random forests survival analyses selected top variables in each category. Top variable selected were shown in graph. (a) Cytoskeleton. (b) Cell adhesion. (c) Extracellular matrix (ECM). (d) Matrix metalloprotease (MMP). The gene symbols with the top variables by the random forests survival analysis are presented in the graphs. (PDF) [file pone.0251272.s003.pdf]

S3 Fig

Takashima et al

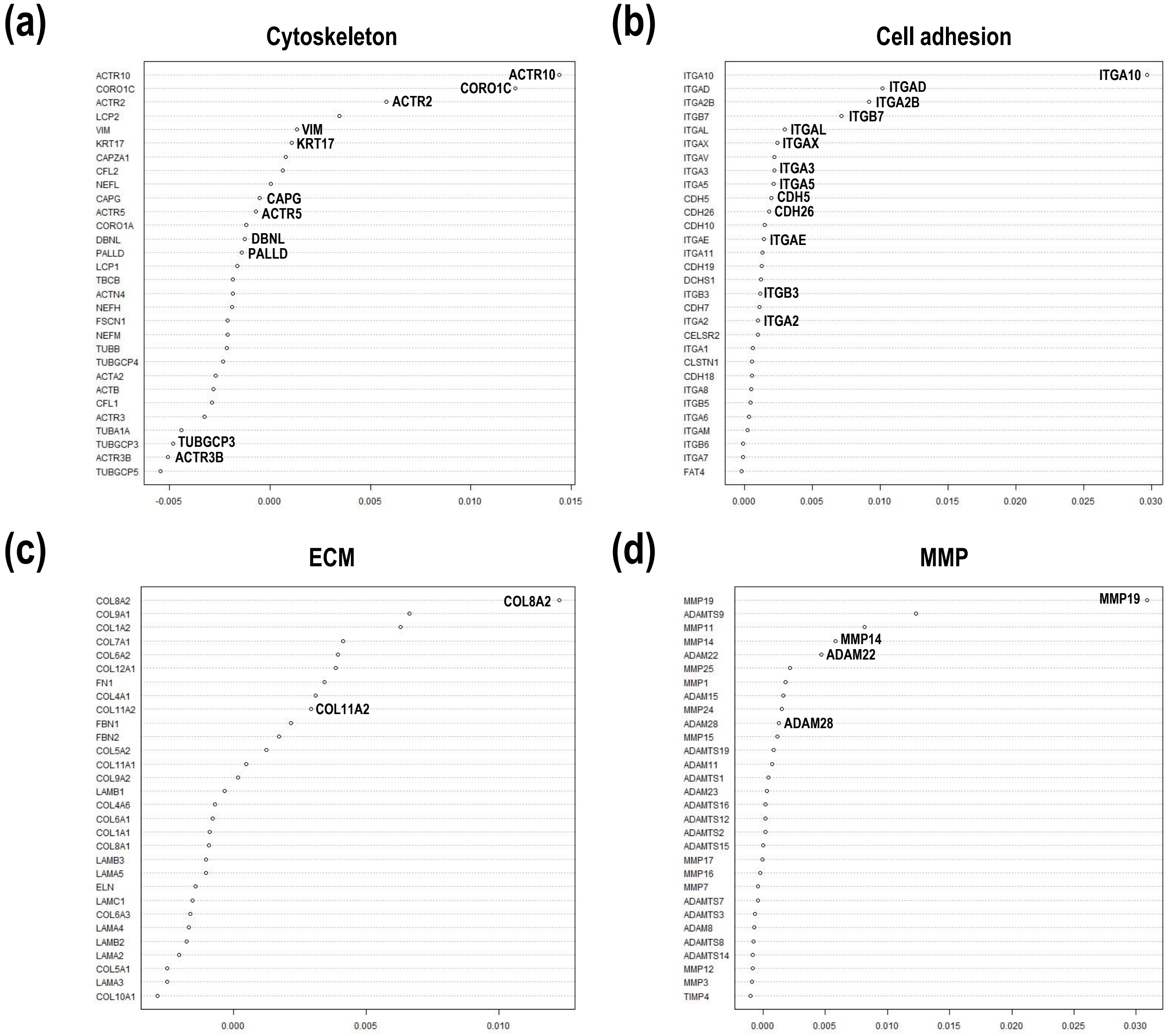

S3 Fig. Variable importance of the genes related to tumor morphology and microenvironment of PCNSL. Random forests survival analyses selected top variables in each category. Top variable selected were shown in graph. (**a**) Cytoskeleton. (**b**) Cell adhesion. (**c**) Extracellular matrix (ECM). (**d**) Matrix metalloprotease (MMP). The gene symbols with the top variables by the random forests survival analysis are presented in the graphs.
