# Supplementary material for: Survival prediction based on the gene expression associated with cancer morphology and microenvironment in primary central nervous system lymphoma
Source: PLoS One. 2021 Jun 24;16(6):e0251272. doi: 10.1371/journal.pone.0251272 (PMC8224980; doi:10.1371/journal.pone.0251272)
Supplement: S1 Table — (PDF) [file pone.0251272.s004.pdf]

S1 Table. Characteristics of the PCNSL patients enrolled in the study.

**age (year)**	
median	67
ave	64.2
min-max	31-85
age>=60	19
age<60	12
**gender**	
female	15
male	16
**KPS**	
median	60
ave	62.5
min-max	40-90
KPS>70	12
KPS<60	19
**LDH**	
median	207
ave	234.7
min-max	132-407
LDH>=216	13
LDH<216	16
**focal lesion**	
single	15
multiple	10
**deep contact**	
yes	19
no	12
**OS (month)**	
median	32
ave	47.5
min-max	5.66-212.6
**censored**	
alive	12
death	19
**GCB subtype**	
GCB	1
non-GCB	24
**MSKCC**	
median	3
ave	2.4
min-max	1-3
**IELSG**	
median	3
ave	2.5
min-max	0-5
**chemotherapy**	
HD-MTX	19
IR	3
polychemotherapy	9
