# Supplementary material for: Survival prediction based on the gene expression associated with cancer morphology and microenvironment in primary central nervous system lymphoma
Source: PLoS One. 2021 Jun 24;16(6):e0251272. doi: 10.1371/journal.pone.0251272 (PMC8224980; doi:10.1371/journal.pone.0251272)
Supplement: S2 Table — (PDF) [file pone.0251272.s005.pdf]

S2 Table. Information for the R version and attached packages used in the study.

```
R version 4.0.2 (2020-06-22)
Platform: x86_64-w64-mingw32/x64 (64-bit)
Running under: Windows 10 x64 (build 18363)

Matrix products: default

locale:
[1] LC_COLLATE=Japanese_Japan.932  LC_CTYPE=Japanese_Japan.932    LC_MONETARY=Japanese_Japan.932 LC_NUMERIC=C
LC_TIME=Japanese_Japan.932

attached base packages:
[1] stats     graphics  grDevices utils     datasets  methods   base

other attached packages:
 [1] msma_2.1                mvtnorm_1.1-1           rpart.plot_3.0.8        rpart_4.1-15            randomForestSRC_2.9.3     PerformanceAnalytics_2.0.4
xts_0.12-0              zoo_1.8-8
 [9] correlation_0.3.0       qgraph_1.6.5            Heatplus_2.34.0         survplot_0.0.7          survival_3.2-3          finalfit_1.0.1          CRUF_0.5.1
flextable_0.5.10
[17] officer_0.3.12          tangram_0.7.1           knitr_1.29              magrittr_1.5            R6_2.4.1                tableone_0.11.1         xlsx_0.6.3

loaded via a namespace (and not attached):
 [1] colorspace_1.4-1    rjson_0.2.20        ellipsis_0.3.1      htmlTable_2.0.0     corpcor_1.6.9       parameters_0.8.0    base64enc_0.1-3     rstudioapi_0.11
mice_3.9.0          lavaan_0.6-6        xml2_1.3.2
[12] splines_4.0.2       mnormt_2.0.0        glasso_1.11         Formula_1.2-3       rJava_0.9-12        broom_0.5.6         cluster_2.1.0       png_0.1-7
miceadds_3.9-14     aod_1.3.1           effectsize_0.3.1
[23] compiler_4.0.2      backports_1.1.7     Matrix_1.2-18       survey_4.0          acepack_1.4.1       htmltools_0.5.0     tools_4.0.2         igraph_1.2.5
gtable_0.3.0        glue_1.4.1          reshape2_1.4.4
[34] dplyr_1.0.0         Rcpp_1.0.4.6        vctrs_0.3.1         nlme_3.1-148        psych_1.9.12.31     insight_0.8.5       xfun_0.15           stringr_1.4.0
xlsxjars_0.6.1      lifecycle_0.2.0     gtools_3.8.2
[45] MASS_7.3-51.6       scales_1.1.1        BDgraph_2.62        parallel_4.0.2      huge_1.3.4.1        RColorBrewer_1.1-2  pbapply_1.4-2       gridExtra_2.3
ggplot2_3.3.2       gdtools_0.2.2       latticeExtra_0.6-29
[56] stringi_1.4.6       bayestestR_0.7.0    checkmate_2.0.0     boot_1.3-25         zip_2.0.4           rlang_0.4.6         pkgconfig_2.0.3     systemfonts_0.2.3
d3Network_0.5.2.1   evaluate_0.14       lattice_0.20-41
[67] purrr_0.3.4         htmlwidgets_1.5.1   tidyselect_1.1.0    plyr_1.8.6          generics_0.0.2      Hmisc_4.4-0         DBI_1.1.0           pillar_1.4.4
whisker_0.4         foreign_0.8-80      abind_1.4-5
[78] nnet_7.3-14         tibble_3.0.1        crayon_1.3.4        uuid_0.1-4          fdrtool_1.2.15      tmvnsim_1.0-2       rmarkdown_2.3       jpeg_0.1-8.1
grid_4.0.2          data.table_1.12.8   pbivnorm_0.6.0
[89] forcats_0.5.0       digest_0.6.25       tidyr_1.1.0         stats4_4.0.2        munsell_0.5.0       quadprog_1.5-8      mitools_2.4
```
