# Supplementary material for: Survival prediction based on the gene expression associated with cancer morphology and microenvironment in primary central nervous system lymphoma
Source: PLoS One. 2021 Jun 24;16(6):e0251272. doi: 10.1371/journal.pone.0251272 (PMC8224980; doi:10.1371/journal.pone.0251272)
Supplement: S3 Table — (PDF) [file pone.0251272.s006.pdf]

S3 Table. A list of the genes analyzed in the study.

Cytoskeleton

Gene symbol	Gene name	Molecule
ACTA2	actin, alpha 2, smooth muscle, aorta(ACTA2)	Actin
ACTB	actin beta(ACTB)	Actin
ACTN4	actinin alpha 4(ACTN4)	Actin
ACTR10	actin-related protein 10 homolog(ACTR10)	Actin
ACTR2	ARP2 actin related protein 2 homolog(ACTR2)	Actin
ACTR3	ARP3 actin related protein 3 homolog(ACTR3)	Actin
ACTR3B	ARP3 actin related protein 3 homolog B(ACTR3B)	Actin
ACTR5	ARP5 actin-related protein 5 homolog(ACTR5)	Actin
CAPG	capping actin protein, gelsolin like(CAPG)	Actin
CAPZA1	capping actin protein of muscle Z-line alpha subunit 1(CAPZA1)	Actin
CFL1	cofilin 1(CFL1)	Cofilin
CFL2	cofilin 2(CFL2)	Cofilin
CORO1A	coronin 1A(CORO1A)	Coronin
CORO1C	coronin 1C(CORO1C)	Coronin
DBNL	drebrin like(DBNL)	Drebrin
DES	desmin(DES)	Desmin
FSCN1	fascin actin-bundling protein 1(FSCN1)	Fascin
GFAP	glial fibrillary acidic protein(GFAP)	GFAP
KRT17	keratin 17(KRT17)	Cytokeratin
LCP1	lymphocyte cytosolic protein 1(LCP1)	Plastin
LCP2	lymphocyte cytosolic protein 2(LCP2)	Plastin
NEFH	neurofilament heavy polypeptide(NEFH)	Neurofilament
NEFL	neurofilament, light polypeptide(NEFL)	Neurofilament
NEFM	neurofilament, medium polypeptide(NEFM)	Neurofilament
PALLD	palladin, cytoskeletal associated protein(PALLD)	Palladin
TBCB	tubulin folding cofactor B(TBCB)	Tubulin
TUBA1A	tubulin alpha 1a(TUBA1A)	Tubulin
TUBB	tubulin beta class I(TUBB)	Tubulin
TUBGCP3	tubulin gamma complex associated protein 3(TUBGCP3)	Tubulin
TUBGCP4	tubulin gamma complex associated protein 4(TUBGCP4)	Tubulin
TUBGCP5	tubulin gamma complex associated protein 5(TUBGCP5)	Tubulin
VIM	vimentin(VIM)	Vimentin

Cell adhesion

Gene symbol	Gene name	Molecule
CDH1	cadherin 1(CDH1)	E-cadherin (epithelial)
CDH10	cadherin 10(CDH10)	Type 2 (T2-cadherin)
CDH11	cadherin 11(CDH11)	OB-cadherin (osteoblast)
CDH12	cadherin 12(CDH12)	Type 2 (N-cadherin 2)
CDH13	cadherin 13(CDH13)	T-cadherin/H-cadherin (heart)
CDH15	cadherin 15(CDH15)	M-cadherin (myotubule)
CDH16	cadherin 16(CDH16)	KSP-cadherin
CDH17	cadherin 17(CDH17)	LI cadherin (liver-intestine)
CDH18	cadherin 18(CDH18)	Cadherin, type 2
CDH19	cadherin 19(CDH19)	Cadherin, type 2
CDH2	cadherin 2(CDH2)	N-cadherin (neural)
CDH20	cadherin 20(CDH20)	Cadherin, type 2
CDH22	cadherin 22(CDH22)	Cadherin, ungrouped
CDH23	cadherin related 23(CDH23)	Neurosensory epithelium
CDH24	cadherin 24(CDH24)	Cadherin, ungrouped
CDH26	cadherin 26(CDH26)	Cadherin, ungrouped
CDH3	cadherin 3(CDH3)	P-cadherin (placental)
CDH4	cadherin 4(CDH4)	R-cadherin (retinal)
CDH5	cadherin 5(CDH5)	VE-cadherin (vascular endothelial)
CDH6	cadherin 6(CDH6)	K-cadherin (kidney)
CDH7	cadherin 7(CDH7)	Cadherin, type 2
CDH8	cadherin 8(CDH8)	Cadherin, type 2
CDH9	cadherin 9(CDH9)	Type 2 (T1-cadherin)
CELSR1	cadherin EGF LAG seven-pass G-type receptor 1(CELSR1)	EGF/LAG seven-pass G-type receptor
CELSR2	cadherin EGF LAG seven-pass G-type receptor 2(CELSR2)	EGF/LAG seven-pass G-type receptor
CELSR3	cadherin EGF LAG seven-pass G-type receptor 3(CELSR3)	EGF/LAG seven-pass G-type receptor
CLSTN1	calsyntenin 1(CLSTN1)	Calsyntenin
CLSTN2	calsyntenin 2(CLSTN2)	Calsyntenin
CLSTN3	calsyntenin 3(CLSTN3)	Calsyntenin
DCHS1	dachsous cadherin-related 1(DCHS1)	Dachsous
DCHS2	dachsous cadherin-related 2(DCHS2)	Dachsous
DSC1	desmocollin 1(DSC1)	Desmocollin
DSC2	desmocollin 2(DSC2)	Desmocollin
DSC3	desmocollin 3(DSC3)	Desmocollin
DSG1	desmoglein 1(DSG1)	Desmoglein
DSG2	desmoglein 2(DSG2)	Desmoglein
DSG3	desmoglein 3(DSG3)	Desmoglein
DSG4	desmoglein 4(DSG4)	Desmoglein
FAT2	FAT atypical cadherin 2(FAT2)	FAT
FAT4	FAT atypical cadherin 4(FAT4)	FAT
ITGA1	integrin subunit alpha 1(ITGA1)	Integrin
ITGA10	integrin subunit alpha 10(ITGA10)	Integrin
ITGA11	integrin subunit alpha 11(ITGA11)	Integrin
ITGA2	integrin subunit alpha 2(ITGA2)	Integrin
ITGA2B	integrin subunit alpha 2b(ITGA2B)	Integrin
ITGA3	integrin subunit alpha 3(ITGA3)	Integrin
ITGA4	integrin subunit alpha 4(ITGA4)	Integrin
ITGA5	integrin subunit alpha 5(ITGA5)	Integrin
ITGA6	integrin subunit alpha 6(ITGA6)	Integrin
ITGA7	integrin subunit alpha 7(ITGA7)	Integrin
ITGA8	integrin subunit alpha 8(ITGA8)	Integrin
ITGA9	integrin subunit alpha 9(ITGA9)	Integrin
ITGAD	integrin subunit alpha D(ITGAD)	Integrin
ITGAE	integrin subunit alpha E(ITGAE)	Integrin
ITGAL	integrin subunit alpha L(ITGAL)	Integrin
ITGAM	integrin subunit alpha M(ITGAM)	Integrin
ITGAV	integrin subunit alpha V(ITGAV)	Integrin
ITGAX	integrin subunit alpha X(ITGAX)	Integrin
ITGB1	integrin subunit beta 1(ITGB1)	Integrin
ITGB2	integrin subunit beta 2(ITGB2)	Integrin
ITGB3	integrin subunit beta 3(ITGB3)	Integrin
ITGB4	integrin subunit beta 4(ITGB4)	Integrin
ITGB5	integrin subunit beta 5(ITGB5)	Integrin
ITGB6	integrin subunit beta 6(ITGB6)	Integrin
ITGB7	integrin subunit beta 7(ITGB7)	Integrin
ITGB8	integrin subunit beta 8(ITGB8)	Integrin
RET	ret proto-oncogene(RET)	Ret proto-oncogene

S3 Table. A list of the genes analyzed in the study.

Extracellular matrix

Gene symbol	Gene name	Molecule
COL10A1	collagen type X alpha 1 chain(COL10A1)	Collagen
COL11A1	collagen type XI alpha 1 chain(COL11A1)	Collagen
COL11A2	collagen type XI alpha 2 chain(COL11A2)	Collagen
COL12A1	collagen type XII alpha 1 chain(COL12A1)	Collagen
COL1A1	collagen type I alpha 1 chain(COL1A1)	Collagen
COL1A2	collagen type I alpha 2 chain(COL1A2)	Collagen
COL2A1	collagen type II alpha 1 chain(COL2A1)	Collagen
COL3A1	collagen type III alpha 1 chain(COL3A1)	Collagen
COL4A1	collagen type IV alpha 1 chain(COL4A1)	Collagen
COL4A6	collagen type IV alpha 6 chain(COL4A6)	Collagen
COL5A1	collagen type V alpha 1 chain(COL5A1)	Collagen
COL5A2	collagen type V alpha 2 chain(COL5A2)	Collagen
COL5A3	collagen type V alpha 3 chain(COL5A3)	Collagen
COL6A1	collagen type VI alpha 1 chain(COL6A1)	Collagen
COL6A2	collagen type VI alpha 2 chain(COL6A2)	Collagen
COL6A3	collagen type VI alpha 3 chain(COL6A3)	Collagen
COL7A1	collagen type VII alpha 1 chain(COL7A1)	Collagen
COL8A1	collagen type VIII alpha 1 chain(COL8A1)	Collagen
COL8A2	collagen type VIII alpha 2 chain(COL8A2)	Collagen
COL9A1	collagen type IX alpha 1 chain(COL9A1)	Collagen
COL9A2	collagen type IX alpha 2 chain(COL9A2)	Collagen
COL9A3	collagen type IX alpha 3 chain(COL9A3)	Collagen
ELN	elastin(ELN)	Elastin
FBN1	fibrillin 1(FBN1)	Fibrillin
FBN2	fibrillin 2(FBN2)	Fibrillin
FBN3	fibrillin 3(FBN3)	Fibrillin
FN1	fibronectin 1(FN1)	Fibronectin
LAMA1	laminin subunit alpha 1(LAMA1)	Laminin
LAMA2	laminin subunit alpha 2(LAMA2)	Laminin
LAMA3	laminin subunit alpha 3(LAMA3)	Laminin
LAMA4	laminin subunit alpha 4(LAMA4)	Laminin
LAMA5	laminin subunit alpha 5(LAMA5)	Laminin
LAMB1	laminin subunit beta 1(LAMB1)	Laminin
LAMB2	laminin subunit beta 2(LAMB2)	Laminin
LAMB3	laminin subunit beta 3(LAMB3)	Laminin
LAMB4	laminin subunit beta 4(LAMB4)	Laminin
LAMC1	laminin subunit gamma 1(LAMC1)	Laminin
LAMC2	laminin subunit gamma 2(LAMC2)	Laminin
LAMC3	laminin subunit gamma 3(LAMC3)	Laminin

Matrix metalloproteinase

Gene symbol	Gene name	Molecule
ADAM10	ADAM metallopeptidase domain 10(ADAM10)	ADAM
ADAM11	ADAM metallopeptidase domain 11(ADAM11)	ADAM
ADAM12	ADAM metallopeptidase domain 12(ADAM12)	ADAM
ADAM15	ADAM metallopeptidase domain 15(ADAM15)	ADAM
ADAM17	ADAM metallopeptidase domain 17(ADAM17)	ADAM
ADAM18	ADAM metallopeptidase domain 18(ADAM18)	ADAM
ADAM19	ADAM metallopeptidase domain 19(ADAM19)	ADAM
ADAM2	ADAM metallopeptidase domain 2(ADAM2)	ADAM
ADAM20	ADAM metallopeptidase domain 20(ADAM20)	ADAM
ADAM21	ADAM metallopeptidase domain 21(ADAM21)	ADAM
ADAM22	ADAM metallopeptidase domain 22(ADAM22)	ADAM
ADAM23	ADAM metallopeptidase domain 23(ADAM23)	ADAM
ADAM28	ADAM metallopeptidase domain 28(ADAM28)	ADAM
ADAM29	ADAM metallopeptidase domain 29(ADAM29)	ADAM
ADAM30	ADAM metallopeptidase domain 30(ADAM30)	ADAM
ADAM33	ADAM metallopeptidase domain 33(ADAM33)	ADAM
ADAM7	ADAM metallopeptidase domain 7(ADAM7)	ADAM
ADAM8	ADAM metallopeptidase domain 8(ADAM8)	ADAM
ADAM9	ADAM metallopeptidase domain 9(ADAM9)	ADAM
ADAMTS1	ADAM metallopeptidase with thrombospondin type 1 motif 1(ADAMTS1)	ADAMTS
ADAMTS10	ADAM metallopeptidase with thrombospondin type 1 motif 10(ADAMTS10)	ADAMTS
ADAMTS12	ADAM metallopeptidase with thrombospondin type 1 motif 12(ADAMTS12)	ADAMTS
ADAMTS13	ADAM metallopeptidase with thrombospondin type 1 motif 13(ADAMTS13)	ADAMTS
ADAMTS14	ADAM metallopeptidase with thrombospondin type 1 motif 14(ADAMTS14)	ADAMTS
ADAMTS15	ADAM metallopeptidase with thrombospondin type 1 motif 15(ADAMTS15)	ADAMTS
ADAMTS16	ADAM metallopeptidase with thrombospondin type 1 motif 16(ADAMTS16)	ADAMTS
ADAMTS17	ADAM metallopeptidase with thrombospondin type 1 motif 17(ADAMTS17)	ADAMTS
ADAMTS18	ADAM metallopeptidase with thrombospondin type 1 motif 18(ADAMTS18)	ADAMTS
ADAMTS19	ADAM metallopeptidase with thrombospondin type 1 motif 19(ADAMTS19)	ADAMTS
ADAMTS2	ADAM metallopeptidase with thrombospondin type 1 motif 2(ADAMTS2)	ADAMTS
ADAMTS20	ADAM metallopeptidase with thrombospondin type 1 motif 20(ADAMTS20)	ADAMTS
ADAMTS3	ADAM metallopeptidase with thrombospondin type 1 motif 3(ADAMTS3)	ADAMTS
ADAMTS4	ADAM metallopeptidase with thrombospondin type 1 motif 4(ADAMTS4)	ADAMTS
ADAMTS5	ADAM metallopeptidase with thrombospondin type 1 motif 5(ADAMTS5)	ADAMTS
ADAMTS6	ADAM metallopeptidase with thrombospondin type 1 motif 6(ADAMTS6)	ADAMTS
ADAMTS7	ADAM metallopeptidase with thrombospondin type 1 motif 7(ADAMTS7)	ADAMTS
ADAMTS8	ADAM metallopeptidase with thrombospondin type 1 motif 8(ADAMTS8)	ADAMTS
ADAMTS9	ADAM metallopeptidase with thrombospondin type 1 motif 9(ADAMTS9)	ADAMTS
MMP1	matrix metallopeptidase 1(MMP1)	Matrix metalloproteinase
MMP10	matrix metallopeptidase 10(MMP10)	Matrix metalloproteinase
MMP11	matrix metallopeptidase 11(MMP11)	Matrix metalloproteinase
MMP12	matrix metallopeptidase 12(MMP12)	Matrix metalloproteinase
MMP13	matrix metallopeptidase 13(MMP13)	Matrix metalloproteinase
MMP14	matrix metallopeptidase 14(MMP14)	Matrix metalloproteinase
MMP15	matrix metallopeptidase 15(MMP15)	Matrix metalloproteinase
MMP16	matrix metallopeptidase 16(MMP16)	Matrix metalloproteinase
MMP17	matrix metallopeptidase 17(MMP17)	Matrix metalloproteinase
MMP19	matrix metallopeptidase 19(MMP19)	Matrix metalloproteinase
MMP2	matrix metallopeptidase 2(MMP2)	Matrix metalloproteinase
MMP20	matrix metallopeptidase 20(MMP20)	Matrix metalloproteinase
MMP21	matrix metallopeptidase 21(MMP21)	Matrix metalloproteinase
MMP23A	matrix metallopeptidase 23A (pseudogene)(MMP23A)	Matrix metalloproteinase
MMP23B	matrix metallopeptidase 23B(MMP23B)	Matrix metalloproteinase
MMP24	matrix metallopeptidase 24(MMP24)	Matrix metalloproteinase
MMP25	matrix metallopeptidase 25(MMP25)	Matrix metalloproteinase
MMP26	matrix metallopeptidase 26(MMP26)	Matrix metalloproteinase
MMP27	matrix metallopeptidase 27(MMP27)	Matrix metalloproteinase
MMP28	matrix metallopeptidase 28(MMP28)	Matrix metalloproteinase
MMP3	matrix metallopeptidase 3(MMP3)	Matrix metalloproteinase
MMP7	matrix metallopeptidase 7(MMP7)	Matrix metalloproteinase
MMP8	matrix metallopeptidase 8(MMP8)	Matrix metalloproteinase
MMP9	matrix metallopeptidase 9(MMP9)	Matrix metalloproteinase
TIMP1	TIMP metallopeptidase inhibitor 1(TIMP1)	Metalloproteinase inhibitor
TIMP2	TIMP metallopeptidase inhibitor 2(TIMP2)	Metalloproteinase inhibitor
TIMP3	TIMP metallopeptidase inhibitor 3(TIMP3)	Metalloproteinase inhibitor
TIMP4	TIMP metallopeptidase inhibitor 4(TIMP4)	Metalloproteinase inhibitor
