# Supplementary material for: Survival prediction based on the gene expression associated with cancer morphology and microenvironment in primary central nervous system lymphoma
Source: PLoS One. 2021 Jun 24;16(6):e0251272. doi: 10.1371/journal.pone.0251272 (PMC8224980; doi:10.1371/journal.pone.0251272)
Supplement: S4 Table — (PDF) [file pone.0251272.s007.pdf]

S4 Table

Takashima et al

S4 Table. Multivariable risk factors from cox hazard model in tumor morphology and microenvironment of PCNSL.

								Dependent: Surv(OS..month. , censor)	
Category	rowname	coef	exp(coef)	se(coef)	z	p	Mean (SD)	HR (univariable)	HR (multivariable)
Cytoskeleton	ACTA2	1.223	3.396	0.492	2.483161	0.013	4.7 (0.8)	1.39 (0.78-2.48, p=0.267)	3.40 (1.29-8.91, p=0.013)
Cytoskeleton	ACTR10	-2.813	0.06	0.715	-3.933984	<0.001	4.8 (0.6)	0.31 (0.11-0.81, p=0.018)	0.06 (0.01-0.24, p<0.001)
Cytoskeleton	CAPG	-1.131	0.323	0.399	-2.831941	0.005	6.7 (1.1)	1.07 (0.66-1.73, p=0.777)	0.32 (0.15-0.71, p=0.005)
Cytoskeleton	CORO1C	2.428	11.335	0.654	3.712566	<0.001	5.8 (0.6)	1.79 (0.78-4.12, p=0.168)	11.33 (3.15-40.84, p<0.001)
Cytoskeleton	KRT17	7.067	1172.455	1.717	4.115517	<0.001	0.1 (0.2)	4.54 (0.90-22.94, p=0.067)	1172.46 (40.50-33940.27, p<0.001)
Cytoskeleton	PALLD	-1.701	0.182	0.493	-3.449523	0.001	3.2 (1.0)	0.74 (0.42-1.33, p=0.314)	0.18 (0.07-0.48, p=0.001)
Cell adhesion	CDH10	226.09	1.55E+98	5.724	39.49592	<0.001	0.4 (0.6)	0.30 (0.08-1.10, p=0.069)	1.55E+98 (2.08E+93-1.15E+103, p<0.001)
Cell adhesion	CDH18	-92.827	0.00E+00	14.072	-6.59649	<0.001	0.5 (1.0)	0.50 (0.22-1.14, p=0.099)	0.00 (0.00-0.00, p<0.001)
Cell adhesion	CDH5	687.015	2.33E+298	14.889	46.14216	<0.001	1.5 (0.5)	1.84 (0.83-4.09, p=0.135)	2.33E+298 (4.93E+285-Inf, p<0.001)
Cell adhesion	CLSTN1	-369.158	0.00E+00	2.335	-158.08227	<0.001	4.6 (0.4)	0.31 (0.09-1.01, p=0.052)	0.00 (0.00-0.00, p<0.001)
Cell adhesion	FAT4	-818.233	0.00E+00	53.204	-15.37912	<0.001	0.6 (0.4)	0.49 (0.12-1.93, p=0.308)	0.00 (0.00-0.00, p<0.001)
Cell adhesion	ITGA10	259.949	7.84E+112	0.901	288.468	<0.001	1.0 (0.6)	2.41 (0.91-6.38, p=0.076)	7.84E+112 (1.34E+112-4.59E+113, p<0.001)
Cell adhesion	ITGA8	-337.725	0.00E+00	8.343	-40.48082	<0.001	0.3 (0.3)	0.23 (0.04-1.35, p=0.103)	0.00 (0.00-0.00, p<0.001)
Cell adhesion	ITGAE	-209.772	0.00E+00	1.382	-151.77744	<0.001	5.3 (0.8)	0.96 (0.52-1.75, p=0.891)	0.00 (0.00-0.00, p<0.001)
Cell adhesion	ITGAM	-100.931	0.00E+00	3.533	-28.5687	<0.001	3.0 (0.9)	1.12 (0.66-1.90, p=0.675)	0.00 (0.00-0.00, p<0.001)
Cell adhesion	ITGAX	89.053	4.74E+38	1.521	58.53615	<0.001	3.8 (1.3)	1.34 (0.93-1.94, p=0.115)	4.74E+38 (2.40E+37-9.34E+39, p<0.001)
Cell adhesion	ITGB6	261.696	4.50E+113	11.226	23.31243	<0.001	0.1 (0.2)	1.06 (0.10-10.95, p=0.961)	4.50E+113 (1.25E+104-1.62E+123, p<0.001)
Cell adhesion	ITGB7	122.766	2.07E+53	5.63	21.80599	<0.001	4.2 (1.0)	1.62 (0.96-2.76, p=0.073)	2.07E+53 (3.34E+48-1.28E+58, p<0.001)
ECM	COL8A2	0.819	2.269	0.299	2.736546	0.006	2.2 (1.1)	1.65 (1.03-2.66, p=0.038)	2.27 (1.26-4.08, p=0.006)
ECM	FBN1	1.196	3.308	0.54	2.215801	0.027	2.8 (0.6)	1.15 (0.62-2.14, p=0.659)	3.31 (1.15-9.53, p=0.027)
ECM	LAMA2	-1.814	0.163	0.558	-3.249223	0.001	1.5 (0.7)	0.55 (0.27-1.12, p=0.101)	0.16 (0.05-0.49, p=0.001)
ECM	LAMB3	0.927	2.526	0.46	2.013584	0.044	0.9 (0.6)	1.42 (0.64-3.13, p=0.388)	2.53 (1.02-6.23, p=0.044)
MMP	ADAM22	-1.431	0.239	0.492	-2.909649	0.004	2.2 (0.8)	0.35 (0.17-0.72, p=0.005)	0.24 (0.09-0.63, p=0.004)
MMP	ADAM28	0.865	2.375	0.291	2.976034	0.003	4.8 (1.0)	2.16 (1.26-3.70, p=0.005)	2.37 (1.34-4.20, p=0.003)
MMP	MMP11	0.712	2.039	0.361	1.971931	0.049	1.4 (0.8)	1.83 (1.07-3.12, p=0.027)	2.04 (1.00-4.14, p=0.049)
MMP	MMP24	-2.001	0.135	1.004	-1.992022	0.046	0.3 (0.6)	0.33 (0.08-1.39, p=0.132)	0.14 (0.02-0.97, p=0.046)
