# Supplementary material for: Survival prediction based on the gene expression associated with cancer morphology and microenvironment in primary central nervous system lymphoma
Source: PLoS One. 2021 Jun 24;16(6):e0251272. doi: 10.1371/journal.pone.0251272 (PMC8224980; doi:10.1371/journal.pone.0251272)
Supplement: S5 Table — (PDF) [file pone.0251272.s008.pdf]

S5 Table. Summary of the gene signature candidates associated with cancer morphology and microenvironment and the differential expression with MTX resistance in PCNSL.

Differential expression in MTX-resistant PCNSL cell			Contribution to prognosis prediction by statistical analysis		Correlation between gene signature candidate and MTX resistance	
Gene symbol	TKMTX/TK	HKBMLMTX/HKBML	Description		TKMTX	HKBMLMTX
ADAM28	n.s.	upregulated	ADAM28high = poor	Fig 1d		ADAM28
ADAM28	n.s.	upregulated	Input to the prognosis prediction formula with a positive value	Fig 2d		ADAM28
ADAM28	n.s.	upregulated	ADAM28low = good, ADAM28highMMP19 = poor	Fig 3d		ADAM28
ADAM28	n.s.	upregulated	Input to the prognosis prediction formula with a positive value	Fig 5b		ADAM28
CDH18	n.s.	upregulated	Input to the prognosis prediction formula with negative value	Fig 2b		
CDH18	n.s.	upregulated	Input to the prognosis prediction formula with negative value	Fig 5b		
COL11A2	upregulated	upregulated	COL11A2high = poor	Fig 1e	COL11A2	COL11A2
COL8A2	downregulated	n.s.	COL8A2high = poor	Fig 1f		
COL8A2	downregulated	n.s.	Input to the prognosis prediction formula with a positive value	Fig 2c		
COL8A2	downregulated	n.s.	Input to the prognosis prediction formula with a positive value	Fig 5b		
ITGA2	downregulated	n.s.	ITGADlowITGA2low = good	Fig 3b	ITGA2	
ITGAD	n.s.	upregulated	ITGADlowITGA2low = good	Fig 3b	ITGAD	
ITGAD	n.s.	upregulated	ITGADmiddle = poor	Fig 3b		ITGAD
ITGAM	n.s.	upregulated	Input to the prognosis prediction formula with negative value	Fig 2b		
ITGAX	n.s.	upregulated	Input to the prognosis prediction formula with a positive value	Fig 2b		ITGAX
ITGB7	upregulated	n.s.	Input to the prognosis prediction formula with a positive value	Fig 2b	ITGB7	
ITGB7	upregulated	n.s.	ITGB7high = poor (p = 0.073)	Fig S2g	ITGB7	
KRT17	n.s.	upregulated	Input to the prognosis prediction formula with a positive value	Fig 2a		KRT17
KRT17	n.s.	upregulated	Input to the prognosis prediction formula with a positive value	Fig 5b		KRT17
KRT17	n.s.	upregulated	KRT17high = poor (p = 0.067)	Fig S2h		KRT17
MMP11	n.s.	upregulated	MMP11high = poor	Fig 1g		MMP11
MMP11	n.s.	upregulated	Input to the prognosis prediction formula with a positive value	Fig 2d		MMP11
MMP11	n.s.	upregulated	Input to the prognosis prediction formula with a positive value	Fig 5b		MMP11
PALLD	n.s.	upregulated	Input to the prognosis prediction formula with negative value	Fig 2a		

Note: DEG was designated by |log2(fold change)| > 1. n.s., not significant.
